# First-principles and Molecular Dynamics simulation studies of functionalization of Au_32_ golden fullerene with amino acids

**DOI:** 10.1038/s41598-018-29887-5

**Published:** 2018-07-30

**Authors:** M. Darvish Ganji, H. Tavassoli Larijani, R. Alamol-hoda, M. Mehdizadeh

**Affiliations:** 1 Department of Nanochemistry, Faculty of Pharmaceutical Chemistry, Pharmaceutical Sciences Branch, Islamic Azad University (IAUPS), Tehran, Iran; 2 Nanotechnology Research Institute, School of Chemical Engineering, Babol University of Technology, Babol, Iran

## Abstract

With the growing potential applications of nanoparticles in biomedicine especially the increasing concerns of nanotoxicity of gold nanoparticles, the interaction between protein and nanoparticles is proving to be of fundamental interest for bio-functionalization of materials. The interaction of glycine (Gly) amino acid with Au_32_ fullerene was first investigated with B3LYP-D3/TZVP model. Several forms of glycine were selected to better understand the trends in binding nature of glycine interacting with the nanocage. We have evaluated various stable configurations of the Gly/Au_32_ complexes and the calculated adsorption energies and AIM analysis indicate that non-Gly, z-Gly and also tripeptide glycine can form stable bindings with Au_32_ at aqueous solution via their amino nitrogen (N) and/or carbonyl/carboxyl oxygen (O) active sites. Furthermore, cysteine, tyrosine, histidine and phenylalanine amino acids bound also strongly to the Au_32_ nanocage. Electronic structures and quantum molecular descriptors calculations also demonstrate the significant changes in the electronic properties of the nanocage due to the attachment of selected amino acids. DFT based MD simulation for the most stable complex demonstrate that Gly/Au_32_ complex is quite stable at ambient condition. Our *first-principles* findings offer fundamental insights into the functionalization of Au_32_ nanocage and envisage its applicability as novel carrier of the drugs.

## Introduction

Drug delivery has gained a lot of attention as it can minimize the side effects of various drugs. Practically, an ideal drug delivery system is an increasingly effective disease treatment by delivery of a drug to target organs and lowering the side effects as well as sustained release formulations in which the drug is released over a period of time in a controlled manner^1^. In particular, the development of drug delivery strategies has focused on nanoscale particles to improve bioavailability, selectivity and release rate^2–4^.

Nano drug delivery technology has been fully applied in the treatment course of tumor, diabetes, vascular disease and cancer patients, and can provide some guidance for clinical treatment work^5^. Recently, Nanoscience has been used vastly in drug delivery and lots of research groups have been working on this topic for years to make biocompatible and safe drug carriers delivering a handful of dug to predetermined target cells in a controlled release manner^6^.

Various drug delivery systems based on different nanomaterials, such as biodegradable polymers, calcium phosphate, nanoporous silica, Fe_3_O_4_ and carbon based nanomaterials have been investigated in the past decade. Among all of the nanoparticles, gold NPs have gained a lot of attentions since they exhibit anticancer activities along with being a drug carrier. Also, they have interactions with RNA, DNA and other biological molecules^7^. Gold nanoparticles with different shapes and sizes show their unique physical and chemical properties for transporting and unloading the pharmaceuticals. Last but not the least, the gold nanoparticles can be used for hyperthermia since they can absorb near IR and generate heat^8,9^.

However, the gold nanoparticles suffer from low drug loading capacities. Recent discoveries of three dimensional gold based particles, including gold clusters including Au_32_^10,11^, Au_50_^12,13^ and Au_72_^14^ or golden fullerenes^10–14^ have provided new opportunities to use gold based carriers. Apart from the tetrahedral Au_19_ and Au_20_ clusters^15^, a new class of golden cages^16^ has been proposed using both experimental and theoretical methods. Among the three sizes, Au_32_ has been the most intriguing cluster because of its ground state geometry which is an icosahedral golden fullerene. The report showed that the spherically hollow Au_32_ conformation with nearly 0.9 *nm* diameter is structurally similar to C_60_^10^. On the other hand, the ground state Au_32_ geometry has been proposed to be aromatic in nature^17,18^.

The other part of drug delivery involves release time, which strongly depends on carrier and drug interaction. Thus, exploring the interactions between biologically relevant molecules and Au_32_ surface seems to be of crucial importance for designing efficient drug carriers. The predictions from theoretical point of view help the scientists to understand the loading and the release time, loading capacities and even loading and release mechanism. Moreover, knowing the interactions can predict the delivery selectivity and efficiency^19–23^. Meanwhile, since amino acids are the elementary component of biomolecules and can also mimic the chemical properties of complicated biomolecules, it is important to understand the interaction between drug carrier and typical amino acids. Among all the 20 elementary amino acids, glycine might be the best candidate since it is small which makes it possible to investigate their interactions with gold fullerene with a rational computational cost^24–27^. However, as biomolecules such as proteins are much more complicated than the glycine amino acid we have also considered cysteine, tyrosine, histidine and, phenylalanine amino acids which consist amino nitrogen, carboxyl oxygen, carbonyl oxygen, hydroxyl oxygen, imidazole, sulfur and, aromatic rings.

Herein we study the interaction between fullerene**-**like gold nanocage and selected amino acids by using the *first-principles* calculations at the DFT-B3LYP level of theory. DFT calculations have been tremendously applied and shown by several researchers to successfully predict the structural and electronic properties of molecular systems interacting with nanostructured based materials^28–33^. Adsorption energy is calculated and amino acids binding to the gold fullerene nanocage is investigated. We also analyzed the electronic structures and molecular properties for the energetically most favorable complexes. Our results indicate that selected amino acids can form stable bindings to the gold nanocage via their potential active sites. Our theoretical predictions have shed light toward the functionalization of golden nanocage with biomolecules and provide us a rational design and preparation of novel gold**-**based nanomaterials for drug delivery.

## Computational Methodology

The *first-principles* DFT method was employed to calculate the structure and energy of the systems under study. All calculations were performed using the ORCA quantum chemistry code^34^ (version 3.0.0)^35^ at the B3LYP/def2-TZVP^36,37^ level of theory^38,39^. In order to consider the long range non-local interactions, the atom-pairwise dispersion correction (D3)^40,41^ with Becke–Johnson damping (BJ)^42^ was utilized for the systems studied here. The optimized atomic structures of the considered systems were obtained by the density-fitting (resolution-of-the-identity approximation) and chain-of-sphere methods (RIJCOSX)^43^ to accelerate the calculations without loss of accuracy. Furthermore, we utilized the [SD (60, MDF)] effective core potential (ECP) for the Au atoms^44,45^. The nucleus effective charge method was also used for the Au atom. The solvent effects have been described by conductor-like screening models (COSMO)^46^ to consider the electrostatic interaction of molecules with solvent. The adsorption energy was calculated as the difference between the energy of Gly/Au_32_ complexes and the sum of the energies of the corresponding glycine and Au_32_ fullerene (*E*_ads_ = *E*_(Gly/Au32)_ − [E_(Gly)_ + E_(Au32)_]). To eliminate the basis set superposition errors (BSSEs) the Boys–Bernardís counterpoise (CP) scheme^47^ was utilized. It should be noted that positive/negative adsorption energy indicates endothermic/exothermic process, respectively.

We used the quantum mechanical descriptors (QMD) to describe the electronic properties of the glycine molecule and its complexes with the Au_32_ nanocage consisting of ionization potential (IP), electron affinity (EA), global hardness (*η*) and energy gap (*E*g). According to the Koopmans theorem, the highest occupied molecular orbital (HOMO) and the lowest unoccupied molecular orbital (LUMO) energy level are related to the IP and EA, respectively^48^ as follows:

1 $${\rm{IP}}\approx -\,{{\rm{E}}}_{{\rm{HOMO}}}\,{\rm{and}}\,{\rm{EA}}\approx -\,{{\rm{E}}}_{{\rm{LUMO}}}$$

The global hardness is calculated using the relation^49^:

2 $$\eta =({\rm{IP}}-{\rm{EA}})/2$$

The accuracy of our implemented method is validated elsewhere^47,50,51^.

Chemical bonds nature was investigated and characterized using the quantum theory of atoms in molecules (QTAIM) scheme or AIM theory which its theoretical basis has been detailed elsewhere^52–54^. AIM analysis was carried out to deeply understand the interaction natures between Au_32_ fullerene and different amino acids. We used ORCA software to obtain the wave-function used in the bonding analysis at the B3LYP level of theory. The topological analysis, the evaluation of local properties and surface electrostatic potential maxima and minima points were calculated and visualized using the Multiwfn program package^55,56^.

Based on the AIM theory, when two atoms form a bond, a bonding critical point (BCP) appears between the formed bonds. At the BCP, the (ρ(r)) and the sign of its Laplacian determines whether the charge is concentrated as in covalent bonds (∇^2^ρ(r) < 0) or is depleted as in closed shell (electrostatic) interactions (∇^2^ρ(r) > 0). For the shared interactions, the accumulation of electron density can be easily observed across the line that links the involving nuclei whereas for the closed shell interactions the accumulation of charges can be seen at the terminal of interacting nuclei and the BCP in the middle of the bond accounts for the depletion of the electron density.

According to the Bader’s theory^53^, at the BCPs, the total energy density (*H*(*r*_*BCP*_)) is related to the Laplacian of the electron density by the following equation:

3 $$1/4{\nabla }^{2}{\rm{\rho }}\,({r}_{BCP})=G({r}_{BCP})+H({r}_{BCP})$$

where *G*(*r*_*BCP*_) denotes the kinetic energy density which is always a positive value. A pure covalent bond is usually represented by a negative Laplacian and a negative energy density while pure closed shell bonds such as strong hydrogen bonds and ionic bonds are characterized by positive values for Laplacian and energy density. Their intermediated *i*.*e*. partially covalent and highly polar bonds, are classified by positive Laplacian and negative energy density. However, it is noteworthy to mention that there are also exceptions which do not completely follow the aforementioned rules regarding the type of bonds and these criteria should be considered together with the other analysis.

We have further performed *first-principles* MD simulation using the Spanish Initiative for Electronic Simulations with Thousands of Atoms (SIESTA) code^57^ which employs the Born-Oppenheimer dynamics approximation for simulation of molecular systems. To describe the interaction between electrons and ions we utilized the norm-conserving Troullier-Martins pseudopotential^58^ and the generalized gradient approximation (GGA) parameterized by Perdew, Burke and Ernzerhof (PBE)^59^ is employed for the exchange-correlation potential. The localized atomic orbitals for valence wave functions and double-ζ basis sets are employed, with a cutoff energy of 125 Ry for system under study. The Velocity Verlet algorithm was employed for solving the Newton’s equations of motion. The NVT ensemble was used with a Nosé–Hoover thermostat to maintain the temperature at 300 K.

## Results and Discussion

### Interaction between non-ionic and zwitterion glycine and Au_32_

**Figure 1:**
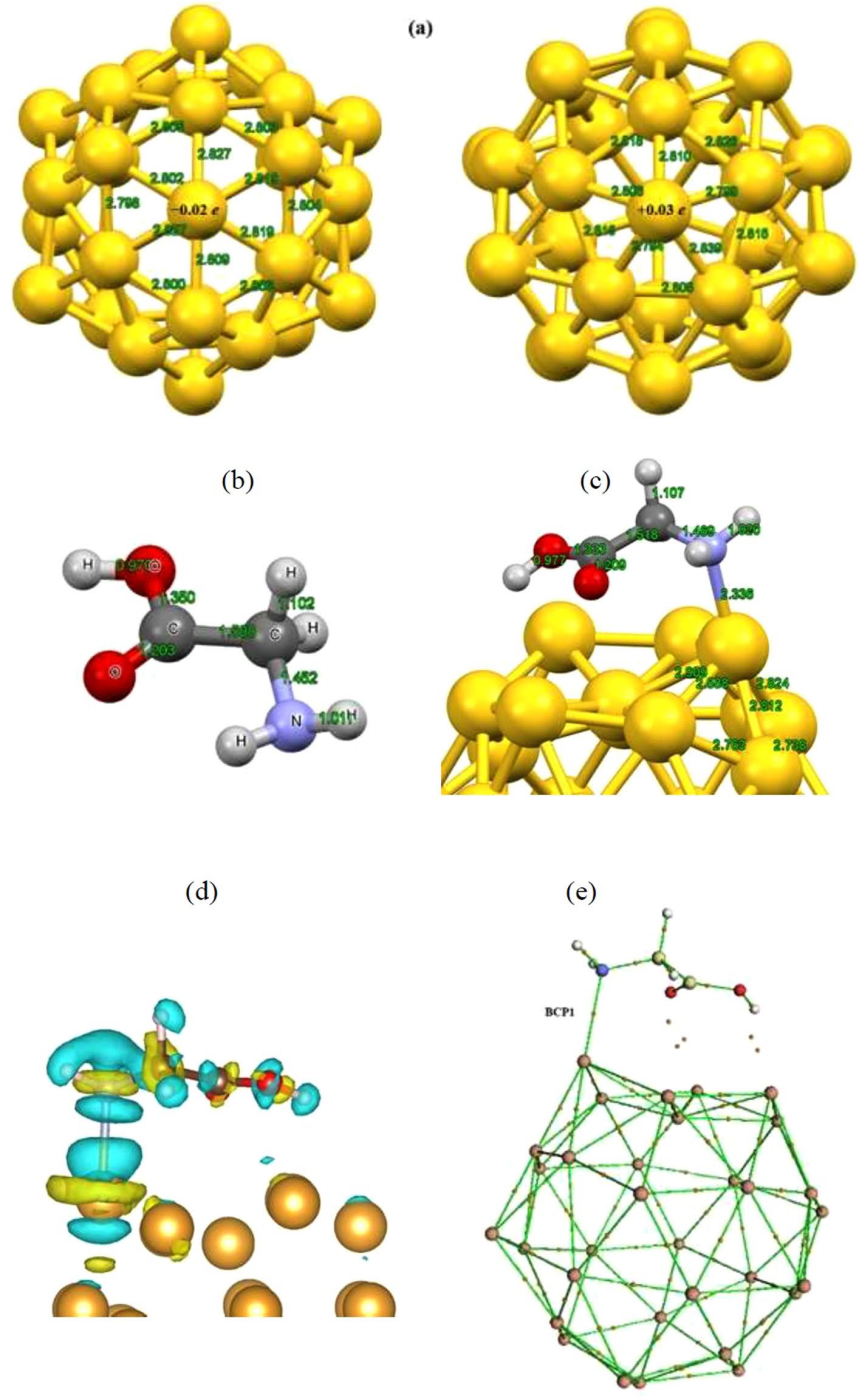
Optimized structures of (**a**) golden fullerene (left: structural parameters for the hexagonal ring - right: structural parameters for the pentagonal ring), (**b**) non-glycine molecule and (**c**) non-Gly/Au_32_ complex in the gas phase obtained with B3LYP-D3-TZVP model. (**d**) The electron density difference plot and (**e**) bonding critical point (BCP) for the formed non-Gly/Au_32_ complex. The white, red, gray, blue and, gold spheres demonstrate the H, O, C, N, and Au atoms, respectively.

We start by describing the equilibrium and electronics structures of golden fullerene and non-ionic glycine (non-Gly) molecules at the gas phase obtained using the B3LYP-D3/TZVP theoretical model. Figure 1 shows the optimized geometries of considered molecules. The average Au–Au bond length in golden fullerene is calculated to be 2.8 Å which agrees well with other theoretical studies^11,60^. Further, the calculated bond lengths of glycine molecule are in good agreement with the literature studied^61^. The charge analysis of optimized geometries depicts various active sites for molecules under consideration. It was found from the atomic charge analysis that the Au atoms placed at the center of pentagon ring (Au-pent) are positively charged hence this half-occupied local state can consequently act as an acceptor entity. This finding reveals that point charges upon the Au_32_ nanocage can improve the binding capability and therefore increase the binding strength between the glycine and the nanocage. On the other hand, the O and N atoms of the glycine are found to be potential electron rich centers and therefore one can expect to glycine molecule attacks to positively charged Au atoms (Au-pent) of golden fullerene via these active sites.

To find the most stable geometries of glycine molecule interacting with golden fullerene, we approached the glycine molecule to the positively charged Au atom (Au-pent) via its N and O active sites. Then, all the considered structures were fully optimized at the B3LYP-D3/TZVP level of theory. The binding energy and equilibrium distance (the closest atoms of the interacting molecules) for energetically favorable complex are determined to be about −1.364 eV (−31.454 kcal/mol) and 2.336 Å, respectively. Figure 1c represents the most stable geometry of non-Gly/Au_32_ complex obtained at the gas phase. As it can be found from the figure, glycine has a significant interaction with the Au atom of pentagon ring through the electronegative N atom. Interaction of glycine with Au_32_ fullerene leads to an increase of the C–N and C–C bonds length of about 0.017 and 0.010 Å in glycine, respectively, while some of the Au–Au bonds length were increased and the others were decreased. Upon interaction, in gold fullerene system, slight deformations in pentagonal and hexagonal rings is observed (deviation of about 27 and 24°, respectively, from the initial conformations). However, it can be seen from the binding distance between nitrogen and Au atom that, this binding distance severely exceeds the sum of the covalent radii of Au and N atoms of about 2.00 Å. Consequently, this bond is unlikely to be of normal covalent nature and there might be other type of interactions which governs the entire bonding process. To understand the adsorption nature, we have represented the charge density difference plot for the formed complex which clearly shows the redistribution of charges upon the attachment of glycine molecule onto the surface of Au_32_. Here, the electron density difference (∆ρ) has been defined as the difference between total charge density of the complex subtracted by sum of the mentioned values for the isolated fragments at the optimized geometry of complex according to the below formula:

4 $${\rm{\Delta }}{\rm{\rho }}={{\rm{\rho }}}_{{\rm{total}}}-({{\rm{\rho }}}_{{\rm{sub}}}+{{\rm{\rho }}}_{{\rm{ads}}})$$

The electron density difference plot for the formed complex is illustrated in Fig. 1d.

The accumulation of electron is represented by yellow color while the electron depletion is shown by blue. It can be seen from the figure that, in the bonding region between N and Au atom, there is no evidence of charge accumulation and the charges mainly accumulate at the terminal regions of the involved atoms in the bonding process. This binding behavior can also partially been explained by considering the geometrical parameters of the amino group of the glycine before and after the adsorption process. It can be seen from the optimized structure of the glycine after the adsorption that the N-C bond length has been increased from 1.452 Å to 1.469 Å. Since the highest occupied molecular orbital (HOMO) of the glycine is mainly located on the nitrogen of the amino group, hence this atom serves as the center of the reactivity and the increase in N-C bond length, facilitates the transfer of electrons from the glycine molecule to the Au_32_ surface. Upon the adsorption of glycine, the HOMO of the adsorbate donates electron to the lowest unoccupied molecular orbital (LUMO) of the substrate which is mainly localized on the pentagon of the Au_32_ molecule in conjunction with some electron back donation to the unoccupied states of the adsorbate molecule as can be seen from the accumulated electrons around the carbon atoms of the glycine. This charge transfer in turn polarizes the substrate and gives rise to the electrostatic interaction between the involved molecules which is expected to plays important role in the attachment of glycine onto the Au_32_ surface.

To further clarify the nature of the formed bond between considered atoms, analysis of the electronic charge density (ρ(r)) and its Laplacian (∇^2^ρ(r)) was carried out using the AIM theory.

Figure 1e illustrates the optimized structure of glycine/Au_32_ complex and the BCPs are shown by orange color. Table 1 lists the calculated results including the Laplacian and energy densities of complex under consideration.

**Table 1:** Calculated Laplacian and energy densities with B3LYP-TZVP model for Glycine/Anionic Glycine/Tripeptide glycine-Au_32_ complexes.

	Glycine		Anionic Glycine		Tripeptide glycine	
	BCP1	BCP1	BCP2	BCP3	BCP1	BCP2
ρ (r)	0.066	0.088	0.057	0.065	0.070	0.053
∇^2^ρ(r)	0.214	0.275	0.228	0.267	0.278	0.213
*H*(r)	−0.011	−0.022	−0.006	−0.009	−0.012	−0.005
G(r)	0.064	0.091	0.063	0.075	0.081	0.058

As can be seen from the Fig. 1e and Table 1, a BCP is evident in the bonding region of nitrogen and Au atom with the ∇^2^ρ value of 0.214 which shows a strong depletion of charge at this critical point and *H*(*r*_*BCP*_) of about −0.011 a.u which shows that there exists an attractive interaction between the two nuclei. Based on these values, this bond has a highly polar nature similar to the situation for coordination and ionic bonds. This is completely in line with the results of charge transfer analysis based on the Hirshfeld method which suggests the transfer of 0.21 *e* from the glycine to the Au_32_ and also the results of charge decomposition analysis (CDA) (not shown here) that the HOMO of the glycine and LUMO of the Au_32_ exhibit the highest contribution to the charge transfer between the two molecules. This corroborates the fact the lone pair of the nitrogen donates electrons to the LUMO of the Au_32_ which resembles a coordination bond rather than a normal covalent bond. These findings reveal a strong interaction between glycine molecule and gold fullerene thus one can conclude that non-Gly/Au_32_ complex is energetically stable at the gas phase.

**Figure 2:**
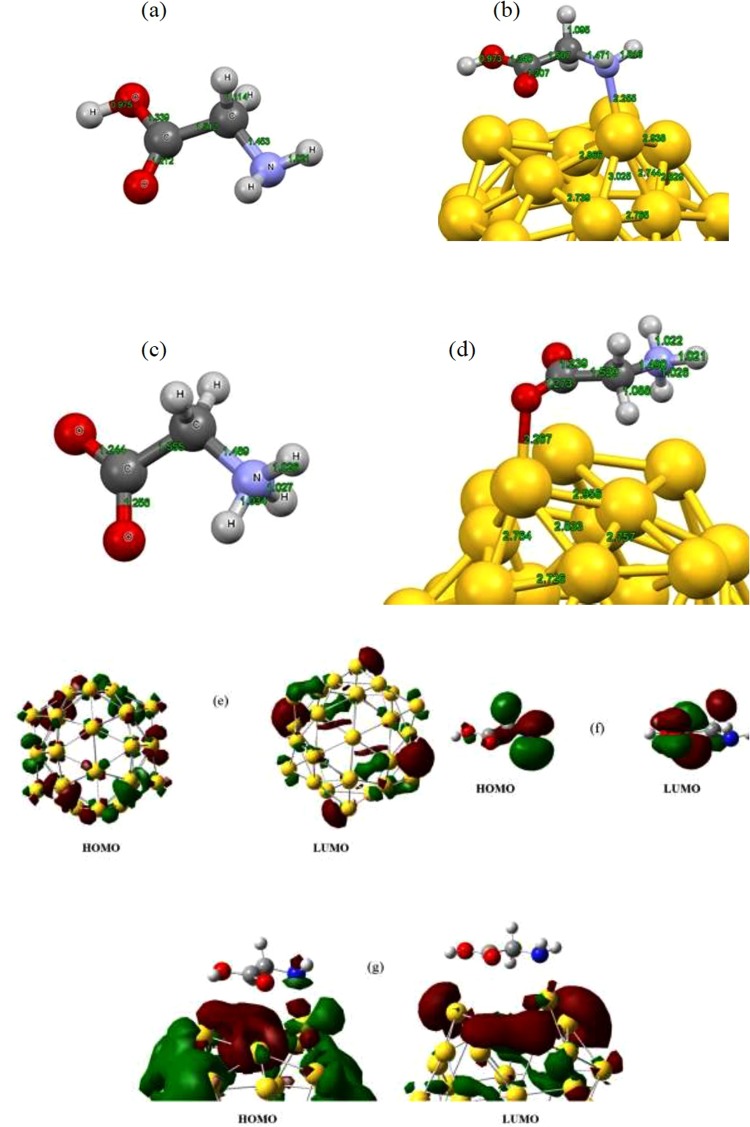
Optimized structures of (**a**) non-glycine molecule, (**b**) non-Gly/Au_32_ complex, (**c**) z-glycine molecule and, (**d**) z-Gly/Au_32_ complex, at aqueous solution obtained with the B3LYP-D3-TZVP level of theory. Calculated profiles of the HOMO and the LUMO frontier orbital depiction of (**e**) Au_32_ nanocage, (**f**) non-Gly and (**g**) non-Gly/Au_32_ complex (Isovalue is taken as 0.02 au).

In order to investigate the influence of solvent on the stability of considered complex we have performed similar calculation procedure for the interacting molecules at aqueous solution. All molecular systems were optimized separately at aqueous solution and then focused on the interaction between two entities. Schematic representation of optimized structure of non-Gly/Au_32_ fullerene complex in aqueous solution is given in Fig. 2. Our *ab initio* results based on the DFT-B3LYP-D3 method with TZVP basis set indicate that considered complex is stable at aqueous solution with the adsorption energy of about −1.444 eV and bonding distance of 2.255 Å. It can be clearly found from the binding information that non-Gly/Au_32_ fullerene complex is energetically more stable in aqueous solution than the gas phase. Furthermore, we have evaluated the binding strength of non-Gly molecule interacting with Au atom placed at the hexagon ring of the Au_32_ nanocage. After full structural optimization on the molecular systems with B3LYP-D3/TZVP model, the binding energy was found to be about −1.40 eV which indicates that non-Gly prefer to be bound to the Au atom of pentagon ring of the nanocage.

We now consider the interaction between zwitterion glycine (z-Gly) amino acid (Fig. 2c) and golden fullerene at aqueous solution. We modeled the z-Gly/Au_32_ nanocage complex so that O atom of carboxyl group was placed above the Au atom of pentagon ring while H atom of NH_3_ group was positioned adjacent to the Au atom of hexagon ring. Full structural optimization was carried out for estimation of adsorption energy for z-Gly/Au_32_ complex. The calculated adsorption energy at the B3LYP-D3/TZVP level showed that z-Gly prefers to be adsorbed at the center of pentagon of golden fullerene via its O atom (see Fig. 2d) with adsorption energy of −1.112 eV and the optimum interacting distance of about 2.267 Å. As a result, the adsorption energy values are highly negative for both forms of glycine amino acid interacting with Au_32_ fullerene suggesting the thermodynamic favorability of respective complexes in aqueous solution though non-Gly counterpart undergoes stronger interaction with the Au_32_ fullerene. Indeed, formation of such complexes seems to be experimentally possible from energetics point of view.

We have further calculated the isosurface corresponding to HOMO and the LUMO states for molecular systems under study (see Fig. 2). In the case of golden fullerene the HOMO state are delocalized on the Au atoms placed at the center of both hexagon and pentagon rings while the LUMO states are mainly localized on the Au atoms of the pentagon center as represented in Fig. 2e. Figure 2f represents the HOMO electronic state to be localized mainly along the amino group with a little contribution on the hydroxyl and carbonyl groups for the non-Gly molecule. The LUMO state obeys however the different trend where the electronic states are localized mainly on the hydroxyl and carbonyl active sites. Upon adsorption of non-Gly amino acid onto Au_32_ fullerene, the electronic state of HOMO isosurface coming mainly from gold nanocage and a little from the glycine while the LUMO isosurface coming only from the gold nanocage (Fig. 2g). This indicates that electronic state contribution towards the adsorption is essentially contributed from golden fullerene supporting electron conduction through this novel media for glycine adsorption.

Furthermore, the energy gap, global reactivity descriptors and dipole moment values have also been calculated for non-ionic/zwitterion glycine-Au_32_ complexes. A comparison of HOMO–LUMO gap and global reactivity descriptors for non-Gly and z-Gly indicate glycine to be quite stable with energy gap around 6.635–7.395 eV and *η* value of 0.146–0.135 eV. The gold fullerene is stable due to its HOMO–LUMO gap of about 2.177 eV and *η* value of 0.459 eV, while Au_32_ fullerene is more reactive than glycine amino acid. Glycine adsorption onto the golden fullerene shows a small decrease in HOMO–LUMO energy gap and increase in *η* value indicating that glycine adsorption renders slightly stability to Au_32_ nanocage. Adsorption of non-Gly onto the Au_32_ fullerene demonstrate more reduction in energy gap value than that of z-Gly counterpart which can be conducive with the rather higher adsorption energy. Furthermore, it was found that the presence of nanocage for loading of glycine facilitates in the increased reactivity of glycine amino acid compared to pristine glycine. The calculated dipole moment which tolerates correlation with adsorption energy values shows that non-Gly/Au_32_ complex has slightly higher dipole moment value than the z-Gly/Au_32_ complex. This finding confirms also the greater extent of adsorption of non-Gly amino acid interacting with Au_32_ fullerene compared to the z-Gly one and also more solubility of the non-Gly/Au_32_ complex rather than other counterpart.

### Interaction between tripeptide glycine and Au_32_

**Figure 3:**
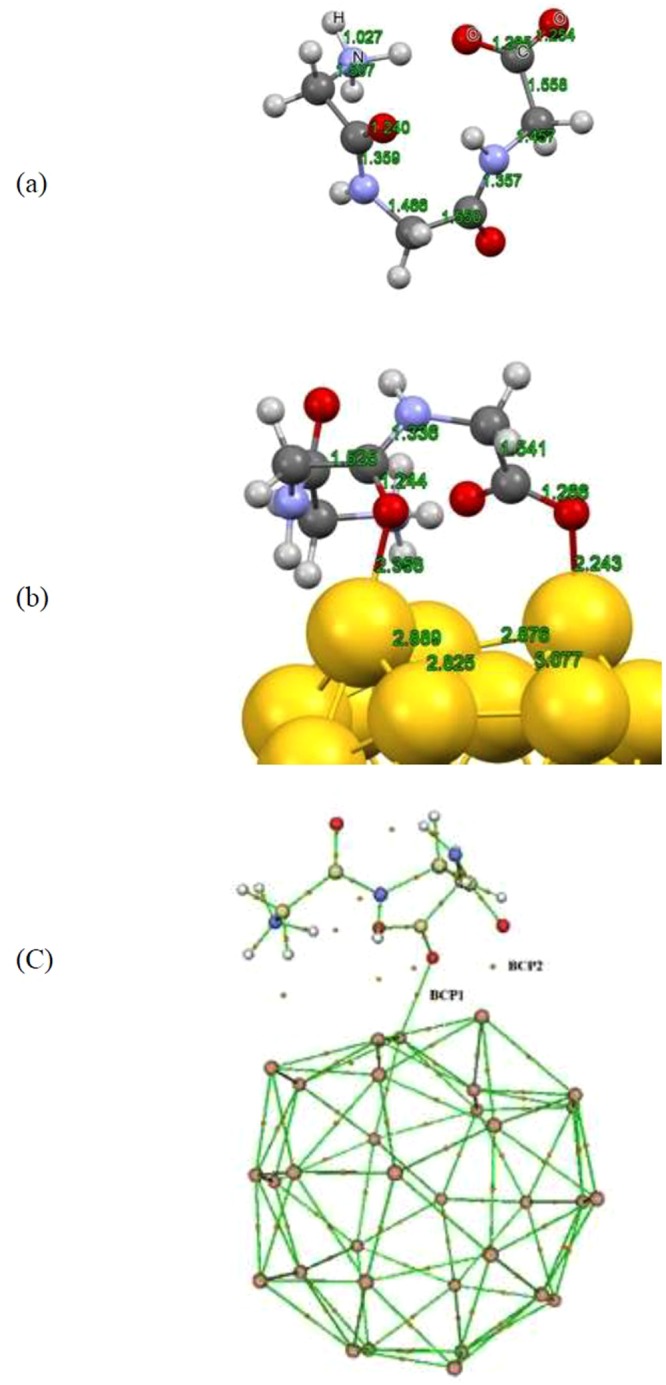
Optimized structures of (**a**) Tripeptide glycine molecule and (**b**) Tripeptide Gly/Au_32_ complex. (**c**) The bonding critical point (BCP) for the formed tripeptide glycine/Au_32_ complex.

To extrapolate the upshots of the current work for larger biological systems that reasonably mimic -for example- the behavior of proteins upon the attachment to Au nanoparticles, we have selected a tripeptide model of the zwitterionic glycine (see Fig. 3) and evaluated the interactions between this biomolecule with Au_32_ nanocage to obtain more realistic results. From the optimized structure of the most stable configuration of tripeptide glycine upon the interaction with Au_32_ fullerene, strong deformations can be observed in the surface of Au_32_ structure together with the formation of bonds between the oxygen atom of the carboxyl and carbonyl groups and the neighboring Au atoms as represented in Fig. 3b. This adsorption configuration accompanies by the release of about −2.058 eV of energy which is high enough to be considered as a stable complex. However, to unveil the nature of the interactions between the two molecules and illustrate the type of the formed bonds, we have performed AIM analysis similar to the previous section and tabulated the results including the Laplacian and energy densities in Table 1. Visualization of the BCPs for the considered complex as shown in Fig. 3c illustrates the appearance of two BCPs within the region of formed bonds which we marked as BCP1 and BCP2. Based on the values in Table 1, both BCPs have positive Laplacian and negative energy densities which represent an intermediate interaction similar to the situation for neutral glycine. However, the magnitude of attractive interactions differs considering the values of energy density for the BCPs. This is in line with the calculated bond lengths where the bond corresponding to the BCP1 has shorter length than that of the BCP2.

The geometrical parameters of the adsorbed amino acid also corroborate the above statements regarding the type of the interactions as the C=O bond lengths where the O-Au bond has been formed are between the range of 1.24 to 1.26 Å which are typical bond lengths for the carbon and oxygen double bond. In this vein, the hybridization of the C=O bond within the region of bond formation did not change which resembles a charge transfer mediated electrostatic interaction together with some structural deformations within the involved molecules. This is consistent with the results of charge transfer analysis which illustrates the transfer of 0.4 *e* from the amino acid to the Au_32_ surface. As a result we could consider the Au_32_ nanocage as a suitable carrier with retained biological potency which made it target for further chemical modification in the drug delivery technology.

### Interaction between ionic glycine and Au_32_

**Figure 4:**
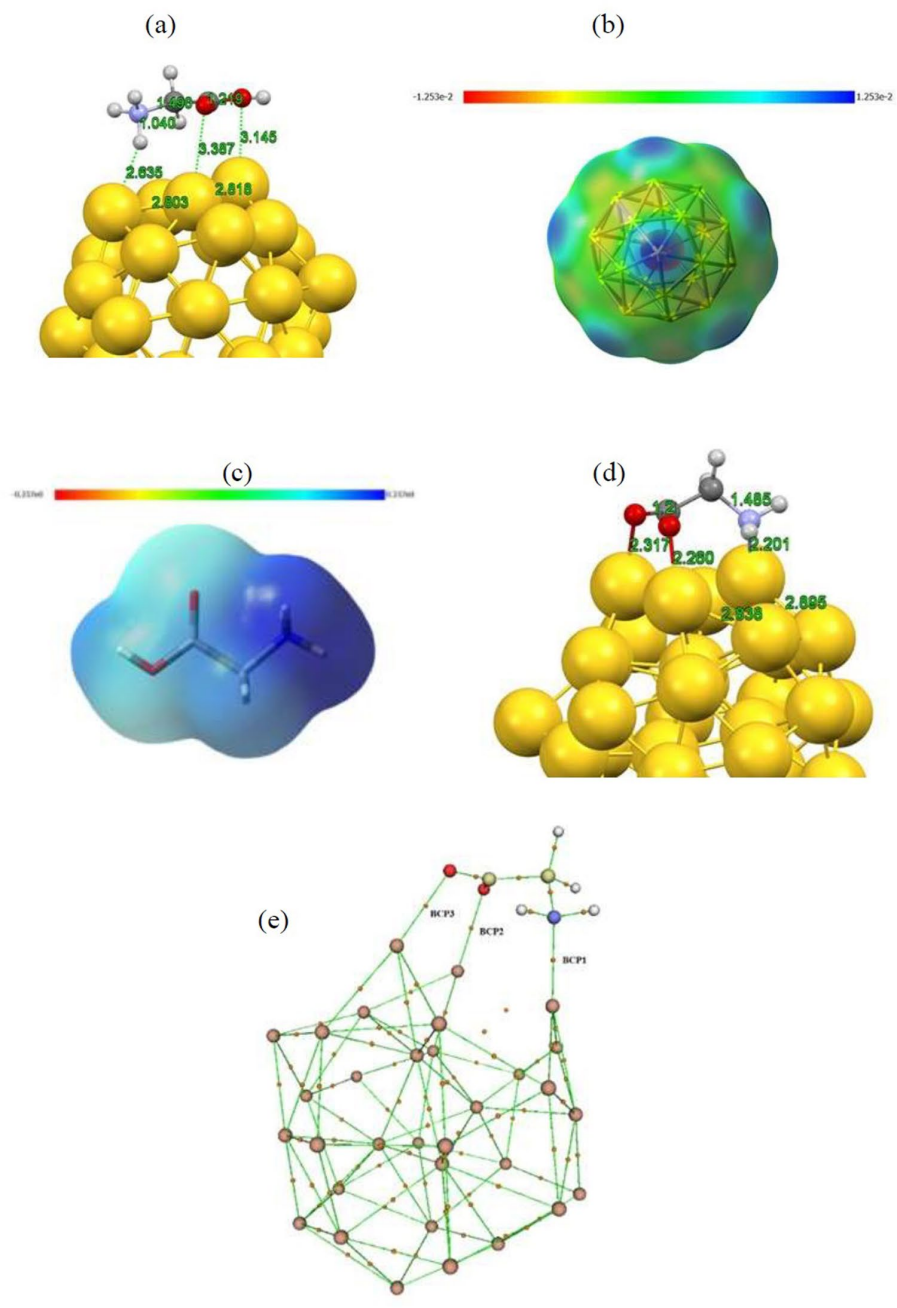
Optimized structures of (**a**) non-glycine molecule, (**b**) non-Gly/Au_32_ complex, (**c**) z-glycine molecule and, (**d**) z-Gly/Au_32_ complex, at aqueous solution obtained with the B3LYP-D3-TZVP level of theory.

The interaction between ionic forms of glycine amino acid and Au_32_ fullerene has also been taken into consideration. For this end, individually optimized structures of anionic and cationic glycine molecule were selected and the stability of the formed glycine/Au_32_ complexes was evaluated through adsorption energy calculations and electronic structure analysis. The optimized structures for the most stable adsorption configurations of cationic and anionic glycine are illustrated in Fig. 4. It can be seen from the figure that the cationic form of glycine does not show any bond formation with the Au_32_ cage skeleton and no structural deformations was seen after the adsorption of cationic form (See Fig. 4b). Indeed, both the Au_32_ and cationic glycine retained their isolated-optimized structures and the cationic form positioned near the Au_32_ surface. This orientation can be easily explained by calculating the molecular electrostatic potential (MEP) maps of the isolated fragments at their optimized geometry as can be observed in Fig. 4b,c. It can be seen from these plots that the local surface maxima are mainly distributed over the pentagon rings of the Au_32_ and the NH_3_ of the cation. Meanwhile, surface minima are mainly located near the hexagonal rings of the Au_32_ while the glycine molecule does not show any surface minima mainly because of its cationic nature. In this regard, the cationic glycine tends to orient above the Au_32_ in a way that its surface maxima approaches close to the surface minima of the Au_32_ in a maximally ESP complementary manner to increase the electrostatic attraction, as can be clearly seen from the optimized structure of the complex system.

The situation on the other hand is slightly different for the anionic glycine where the adsorption of this molecule is accompanied by the release of about −2.699 eV of energy which is considerably higher than that for the cationic counterpart together with strong deformations within the structures of both adsorbate and substrate as can be seen from the Fig. 4d. From the optimized structure it is evident that the glycine anion tilted at the carbon atom which is attached to the amino group with the tilt angle of about 108° after the attachment to the Au_32_ molecule and three bonds have been formed between the carboxyl and amino groups and their adjacent Au atoms. The length of all the formed bonds slightly exceeds the sum of the covalent radii of the respective atoms involved within the bond and these bonds are unlikely to be normal covalent. To clarify the nature of the formed bonds, similar to previous sections AIM analysis was carried out and the results have been given in Table 1. Based on the Laplacian and energy density values for all of the BCPs as shown in Fig. 4e, the formed bonds are within the domain of intermediate bonds as no electron accumulation can be signified from the positive Laplacian while the negative energy densities inform the existence of attractive interactions between the involved atoms of the bonds.

These bonds are mainly governed by one-sided electron donation from the glycine anion to the substrate along with the deformations within the structures of adsorbate and substrate. This electron donation is evident by calculating the transferred charge between the fragments which shows the transfer of 0.8 *e* from the glycine to the Au_32_.

### Interaction of cysteine, histidine, phenylalanine and tyrosine with Au_32_

We now consider other amino acids (cysteine, histidine, phenylalanine and tyrosine) interacting with Au_32_ fullerene and evaluate the binding properties of formed complexes. Similar calculations procedure has been carried out for systems under study. Various orientations considering the potential active sites of interacting molecules such as sulfur and aromatic rings of amino acids and pentagon/hexagon Au atom of golden fullerene have been considered for interacting systems and full structural optimization has been performed followed by the adsorption energy estimation. Our B3LYP-D3/TZVP calculations results demonstrate that all considered amino acids bound strongly to the Au_32_ fullerene surface with adsorption energy of about −1.0 eV (−23.06 kCal/mol).

**Figure 5:**
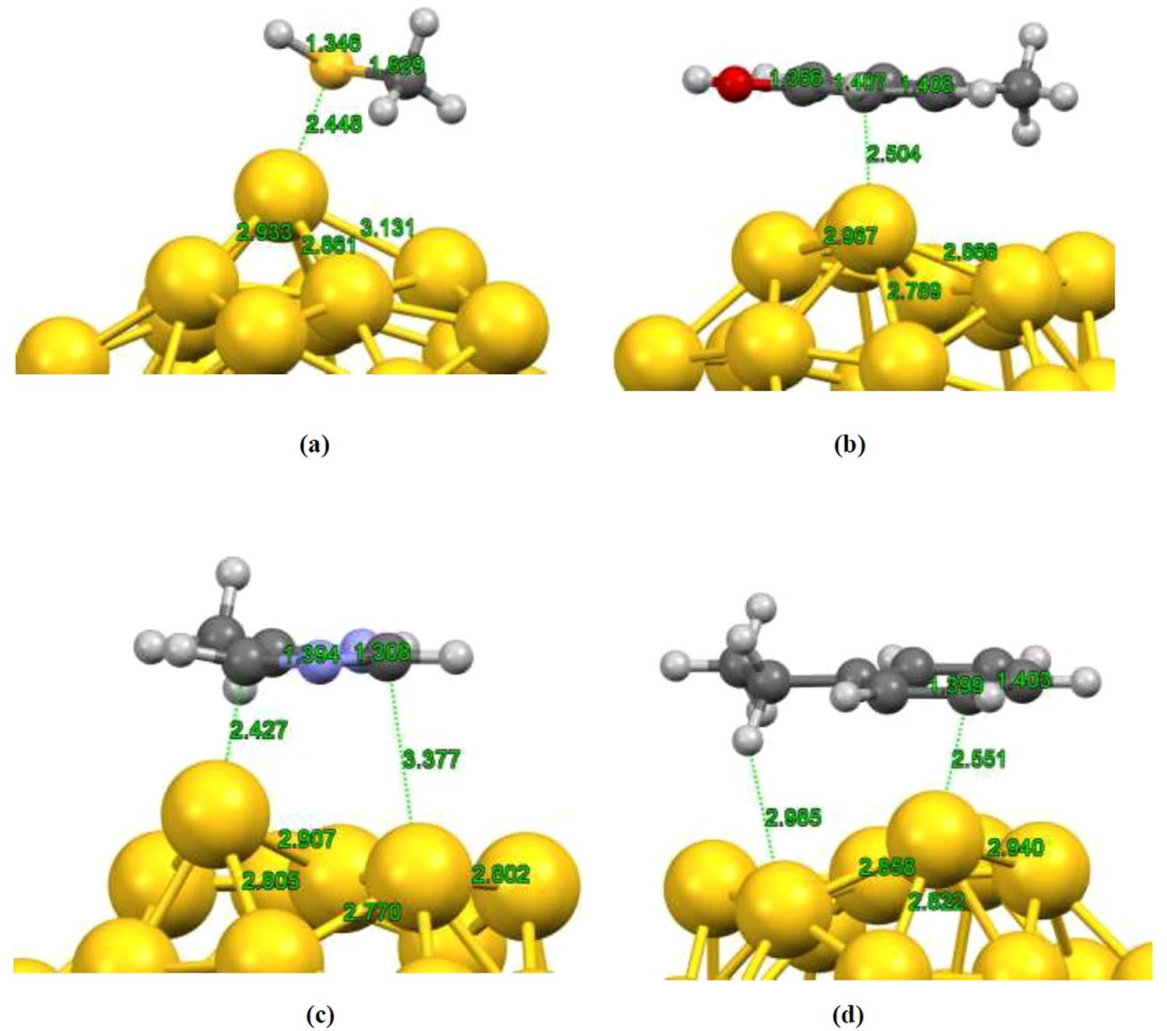
Optimized structure of (**a**) cysteine/Au_32_, (**b**) tyrosine/Au_32_, (**c**) histidine/Au_32_ and (**d**) phenylalanine/Au_32_ complexes.

Cysteine amino acid prefers to bind to the fullerene nanocage through its sulfur atom to the Au atom at the pentagon tip with adsorption energy of −1.172 eV comparable to the Gly/Au_32_ complex. The calculated bonding distance was estimated to be about 2.448 Å which is close to the experimental value of Au-S bond length for the adsorbed thiolates on the Au surface^62^. The optimized geometry of Cys/Au_32_ complex is shown in Fig. 5a. Further investigation about the bond nature of the attached molecule has been carried out by AIM analysis. The result indicated a positive value of 0.187 and negative value of −0.018 for Laplacian and energy density, respectively, which indicates that there exists similar interaction nature (partially covalent and highly polar bonds) for complex under study.

For three other amino acids, our *first-principles* calculations reveal that these molecules were attached to golden fullerene through their C atom in aromatic ring as represented in Fig. 5b–d. The calculated equilibrium distances of Au-C were estimated to be about 2.5 Å which is comparable to similar system (Au dimer adsorbed on the benzene ring) with DFT method at B3LYP/LACVP** level of theory^63^. The AIM analysis for tyrosine/Au_32_ complex demonstrated a similar trend for the Laplacian (0.077) and energy density (−0.005) values indicating similar interacting nature between interacting systems.

The calculated adsorption energies accompanied with charge transfer and optimum distances between interacting systems for more stable configurations are listed in Table 2. As can be seen from the obtained results one can conclude that the interaction natures of selected amino acids are also typical for the chemisorption and the complexes are energetically stable.

**Table 2:** Calculated adsorption energies, charge transfer and bonding distances for cysteine/tyrosine/histidine/phenylalanine-Au_32_ complexes.

Systems	Eads (eV)	Hirshfeld Charge Transfer (*e*)	Bonding Distance (Å)
Cysteine/Au_32_	−1.172	0.281	2.448
Histidine/Au_32_	−1.096	0.217	2.427
Phenylalanine/Au_32_	−1.085	0.165	2.551
Tyrosine/Au_32_	−1.109	0.190	2.504

### *First-principles* molecular dynamics (MD) simulation

**Figure 6:**
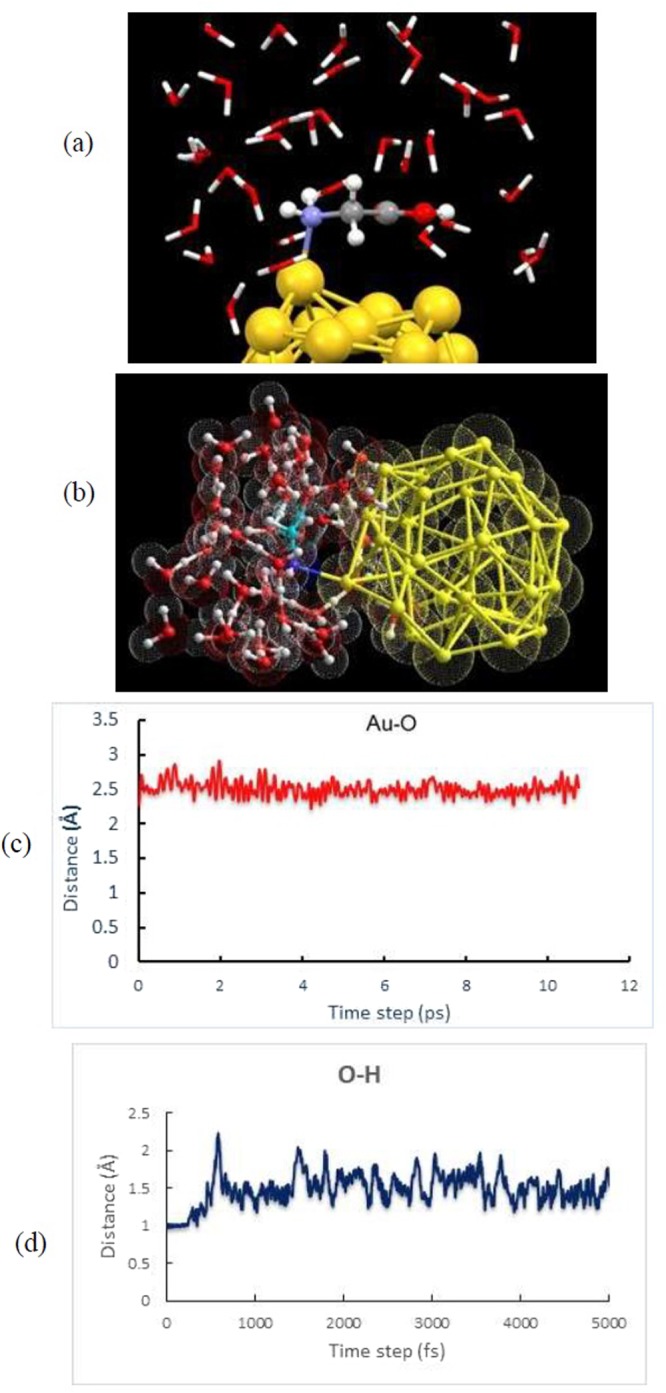
Schematic illustration of (**a**) simulation box filled with 25 water molecules and the energetically favorable configuration of non-Gly/Au_32_ complex and (**b**) snapshot of non-Gly/Au_32_ complex at 10 ps of simulation time. Time evolution of equilibrium bonding distance between (**c**) Au and N atoms of Au_32_ nanocage and glycine molecule and (**d**) H and O atoms of the glycine.

As a rule of thumb the *first-principles* molecular dynamics (MD) simulation has been carried out to assess the binding nature as well as the stability of Gly/Au_32_ complex at ambient condition. MD simulation can reliably explore the global minima state of complex under investigation with realistic simulation of interacting systems by considering explicit water molecules at ambient condition. To this aim, we have further employed an expensive MD simulation based on DFT-D method for the energetically favorable complex (non-Gly/Au_32_) obtained by B3LYP-D3/TZVP model. The simulation system is filled with the non-Gly/Au_32_ complex and 25 water molecules beside the complex as depicted in Fig. 6. We then performed 10 ps of simulation times with 1.0 fs time**-**step in order to obtain the optimized structures of the system under study at ambient conditions.

Our DFT-D based MD simulation results show that the aqueous solution (explicit solvent) affects the stability of glycine isomers and slightly on the geometries of the complex at ambient condition. We found that Gly molecule bound to the Au-pentagon atom via its amino –NH_2_ active site which confirms the current DFT-B3LYP optimization results for glycine molecule attached to the Au atoms of pentagon and hexagon ring. The calculated average value of Au–N bond length (2.504 Å) reveals that the equilibrium bond distance slightly enlarge (the optimized bond length of 2.255 Å). Meanwhile, we observe that the H atom from hydroxyl –OH group of glycine attack to the O atom of water molecule at about 250 fs of simulation time and new bond forms between glycine and water molecules. The average value of O-H bond in glycine was found to be enlarged to 1.386 Å (the initial bond length was 0.973 Å) which indicate that O-H bond was dissociated in the aqueous solution during the simulation time (See Fig. 6c,d). Indeed, one can conclude that the zwitterion glycine isomer is the stable form in the aqueous solution at room temperature. These findings according to the *first-principles* MD simulation indicate that Gly-Au_32_ complex is quite stable and thus it is possible to be used as suitable nanocarrier at ambient condition.

## Conclusions

We have investigated the interaction between Au_32_ fullerene and glycine amino acid with the state-of-the-art DFT calculations at the B3LYP-D3/TZVP level of theory. Various possible orientations have been considered for a glycine molecule attaching to the surface of Au_32_ nanocage. Our DFT calculations at the gas phase demonstrated that non-Gly molecule strongly bound to the outer surface of the nanocage on the top site directly above the Au-pent atom via its amino N active site. The calculated adsorption energy and bonding distance provide sufficient evidence to conclude that the adsorption of glycine onto Au_32_ nanocage is exothermic and the complex is energetically stable. Furthermore, non-Gly amino acid has stronger interaction with Au_32_ fullerene in aqueous solution than the gas phase and the respective complex is energetically more stable. Meanwhile, it was found greater extent of adsorption of non-Gly molecule interacting with Au_32_ nanocage rather than z-Gly counterpart. The calculated electronic structures and charge analysis revealed that the presence of point charges in the Au_32_ nanocage make them suitable candidate for strong binding to glycine amino acid. Furthermore, the global hardness, energy gap and ionization potential of Gly/Au_32_ complex were decreased which increase the reactivity of the respective system.

In order to simulate a realistic model of biomolecule interacting with Au_32_ nanocage we have considered a tripeptide glycine system with zwitterionic structure. Our DFT-D3 based optimization procedure showed that tripeptide glycine bind strongly to the Au_32_ nanocage with binding energy of about −2.0 eV via its carbonyl and carboxyl O atoms active sites. This indicated that tripeptide glycine can form energetically more stable complex rather than both individual non-Gly and z-Gly counterparts. As the rule of thumb for more complicated systems such as proteins we have further evaluated the interaction between Au_32_ nanocage and other amino acids (cysteine, histidine, phenylalanine and tyrosine) including sulfur, imidazole and aromatic groups. Full structural optimization demonstrated that all selected amino acids strongly bound to the Au_32_ cage skeleton with bonding distances of about 2.5 Å and adsorption energy values of about −1.0 eV indicating that the complexes are energetically stable.

Comprehensive DFT based MD simulation at ambient condition has been carried out starting from energetically favorable complex (non-Gly/Au_32_) obtained with B3LYP-D3/TZVP model. The simulation results demonstrated that Gly/Au_32_ complex was quite stable in aqueous solution while non-Gly isomer contribute to a proton transfer phenomenon with adjacent water molecules and thus z-Gly form was found to be the stable isomer in the ambient condition. From the present findings one can predict that proteins which contain hydroxyl oxygen, amino nitrogen and carbonyl oxygen active sites can form stable bindings with Au_32_ fullerene via its potential active sites. Our *first-principles* DFT results provide a well-grounded understanding for the possible formation of complex between bio-molecules and Au_32_ fullerene and can be benchmark for directing experimental efforts for the development of drug delivery at the nanoscale^64–66^.
